# Externally Validated Deep Learning Analysis of Chest Radiographs for Differentiating COVID-19 and Viral Pneumonia

**DOI:** 10.3390/diagnostics16070995

**Published:** 2026-03-26

**Authors:** Michael Masoomi, Latifa Al-Kandari, Haytam Ramzy, Mahday Abass Hamza

**Affiliations:** 1 Department of Radiology, Adan Hospital, Al-Mosely Street, Hadiya 46969, Kuwait; 2 Department of Nuclear Medicine and Molecular Imaging, Adan Hospital, Hadiya 46969, Kuwait; masi755@hotmail.com (L.A.-K.); haytham_rz@hotmail.com (H.R.); mahdyhamza@yahoo.com (M.A.H.)

**Keywords:** chest radiography, deep learning, COVID-19, viral pneumonia, external validation

## Abstract

**Background/Objective:** Chest radiography (CXR) is routinely used in the evaluation of respiratory disease; however, differentiating COVID-19 from other viral pneumonias on CXR remains challenging due to substantial radiographic overlap. In this study, a deep learning-based CXR classification model using a ResNet-50 architecture was developed to categorize images as normal, COVID-19, or non-COVID viral pneumonia, with emphasis on bias mitigation and external validation. **Methods:** Model training and internal validation were performed using harmonized publicly available datasets with patient-level stratified five-fold cross-validation, while generalizability was evaluated using an independent real-world institutional dataset from Adan Hospital, Kuwait, which was excluded from all training, validation, and hyperparameter tuning stages. **Results:** On the public validation dataset (n = 847), the model achieved an overall accuracy of 96.8% with balanced class-wise performance, whereas performance on the independent institutional dataset (n = 320) decreased to 93.7%, consistent with expected domain shift. Calibration analyses demonstrated well-aligned probabilistic estimates on validation data and acceptable calibration on institutional data. Negative predictive values remained high for normal and COVID-19 classes across datasets. Exploratory decision curve analysis demonstrated net benefit patterns for COVID-19 predictions under hypothetical threshold assumptions. **Conclusions:** These findings indicate that, when developed with explicit bias-mitigation strategies and evaluated using independent institutional data, deep learning-based CXR analysis may provide supportive, non-diagnostic decision signals for radiology triage workflows; however, prospective multicenter validation is required prior to clinical adoption.

## 1. Introduction

Chest radiography (CXR) remains one of the most widely used imaging modalities for the evaluation of respiratory disease, particularly in settings where access to advanced imaging such as computed tomography (CT) is limited. Its low cost, rapid acquisition, and broad availability make it central to diagnostic workflows in emergency departments and primary healthcare facilities worldwide [1,2,3]. In Kuwait, chest radiography represents one of the most frequently performed imaging examinations, with over 100,000 adult CXRs performed annually in the Ahmadi Health Area alone [4].

During the COVID-19 pandemic, the surge in imaging demand placed significant strain on radiology services, prompting interest in automated tools that could assist with image interpretation, prioritization, and workflow triage. In this context, deep learning (DL) methods—particularly convolutional neural networks (CNNs)—have demonstrated strong performance across a range of chest X-ray analysis tasks, including abnormality detection and pneumonia classification [5,6,7,8,9,10,11,12,13,14,15]. Recent work has also explored architectures beyond standard CNNs, including graph-based and attention-based models that capture longer-range dependencies and provide additional interpretability. For example, SPX-GNN represents chest X-ray regions as a graph to support relational reasoning for thoracic disease classification [16]. Recent studies have increasingly evaluated deep learning tools for chest radiography in clinically relevant settings, including reader-facing decision support and triage-oriented identification of normal examinations. However, reported performance has varied widely across datasets and institutions, and strong internal results do not necessarily translate to reliable performance under real-world domain shift. Accordingly, independent external testing and probability calibration are increasingly emphasized when proposing AI models intended to support clinical workflows rather than serve as stand-alone diagnostic tools [17,18,19].

However, the differentiation of COVID-19 from other viral pneumonias on chest radiographs remains intrinsically challenging. Radiographic features such as ground-glass opacities, bilateral infiltrates, and consolidation patterns are not disease-specific and may vary with disease stage, patient characteristics, and acquisition parameters [20,21]. Consequently, chest X-ray imaging should not be regarded as a definitive diagnostic test for viral etiology, and AI-based analysis must be framed as a decision-support aid rather than a diagnostic replacement. Accordingly, the present work evaluates multiclass classification performance as a triage-oriented decision-support signal, not as a definitive etiologic diagnosis, and all findings should be interpreted in conjunction with clinical and laboratory context.

A further limitation of many published COVID-19 CXR models relates to dataset bias and shortcut learning. Independent evaluations have demonstrated that models trained on heterogeneous public datasets may exploit non-pathological cues—such as scanner artifacts or institutional markers—rather than genuine disease-related features [18,19,20,21,22,23,24,25,26]. These findings highlight the importance of patient-level partitioning, harmonized preprocessing, and independent validation when developing clinically relevant imaging models [18,24,27]. Accordingly, beyond harmonized preprocessing, we implemented patient-level partitioning and duplicate filtering and reserved a fully independent institutional dataset for final testing; however, these safeguards do not replace targeted shortcut audits and interpretability analyses, which are planned in future work.

Despite extensive COVID-era CXR modeling, relatively few studies have reported external evaluation using locally sourced institutional data from Middle Eastern healthcare systems. Given differences in population case-mix and acquisition practices, this gap limits confidence in transportability. The present work addresses this need by developing a ResNet-50-based multiclass model using harmonized public datasets with patient-level cross-validation and evaluating generalization on an independent institutional cohort from Adan Hospital, Kuwait, complemented by calibration assessment and exploratory decision-analytic evaluation to support a triage-oriented framing.

This study makes the following three main contributions: (i) development of a transparent, bias-aware multiclass CXR model (normal, COVID-19, and non-COVID viral pneumonia) using harmonized public datasets with patient-level cross-validation; (ii) evaluation of generalizability using a strictly held-out real-world institutional dataset from Adan Hospital that was excluded from all training and tuning; and (iii) assessment of probability reliability and decision-support relevance through calibration analysis and exploratory decision curve analysis, supporting a triage-oriented (non-diagnostic) framing.

## 2. Materials and Methods

### 2.1. Study Design and Reporting Framework

This retrospective study developed and evaluated a deep learning-based chest X-ray (CXR) classification model designed to support clinical decision-making through image-level triage. The study design and reporting were guided by the Transparent Reporting of a Multivariable Prediction Model for Individual Prognosis or Diagnosis (TRIPOD) statement [27], which provides recommendations for transparent development, validation, and reporting of prediction models in healthcare. Reporting followed TRIPOD+AI [28] guidelines, adding relevant transparency items. PROBAST+AI [29] risk-of-bias domains were addressed in describing data provenance, domain shift evaluation, and reporting detail.

The overall workflow comprised the following three sequential phases:

(i) model training and internal validation using harmonized publicly available datasets;
(ii) robustness assessment via stratified patient-level cross-validation; and
(iii) independent performance evaluation using a real-world institutional dataset from Adan Hospital, Kuwait.

The institutional dataset was strictly excluded from all stages of model training, cross-validation, and hyperparameter tuning and was reserved exclusively for final testing to assess generalizability under real-world clinical conditions, in accordance with best-practice evaluation guidelines [20].

### 2.2. Data Sources and Dataset Composition

#### 2.2.1. Public Training and Validation Datasets

Four publicly available chest radiography datasets hosted on Kaggle were used to construct the training and validation cohorts. These included the COVID-19 Radiography Database, the COVID-19 Image Data Collection, the NIH ChestX-ray14 dataset, and the RSNA Pneumonia Detection Challenge [30,31,32,33]. These datasets were selected to provide representative samples of the following three diagnostic categories of interest: normal, COVID-19, and non-COVID viral pneumonia. An initial pool of approximately 10,000 images was screened. Images were excluded if they met any of the following criteria:

(i) duplicate images;
(ii) pediatric cases;
(iii) lateral or non-frontal projections;
(iv) poor diagnostic quality (e.g., motion artifact, severe under- or overexposure); or
(v) missing or ambiguous class labels.

Patient-level stratified partitioning and five-fold cross-validation were performed using patient identifiers where available in the source datasets/metadata. For datasets where explicit patient identifiers were unavailable or incomplete, we emphasize that true patient-level separation cannot be guaranteed and that this represents a limitation of public dataset use. In such cases, splitting was performed at the image level using the available metadata fields, and results were interpreted cautiously given the known risk of overlap and hidden duplication in public COVID-19 CXR ecosystems.

Following filtering, 8123 images remained and were divided into training (7276) and validation (847) sets. Each image was categorized as normal, viral pneumonia, or COVID-19. To eliminate dataset bias and prevent shortcut learning, all public datasets underwent identical preprocessing prior to merging, as suggested in the literature [22,23,24,25,26]. Merging and randomization at the patient level took place only after preprocessing to reduce dataset-specific influences.

Additional safeguards included duplicate images filtering prior to cohort construction and splitting to reduce leakage risk across training and validation partitions. Duplicate identification was performed using available metadata-based checks (e.g., filenames and associated fields). We acknowledge that public COVID-19 CXR ecosystems may contain cross-dataset near-duplicates (e.g., re-encoded or resized copies) that are not reliably detectable through metadata alone; explicit near-duplicate auditing is planned for future work.

#### 2.2.2. Institutional Test Dataset (Independent External Evaluation)

An independent institutional dataset was retrospectively collected from the radiology department at Adan Hospital and affiliated primary healthcare centers (PHCs) in Kuwait. From an initial pool of approximately 1000 chest radiographs acquired between January 2022 and December 2023, a total of 320 anonymized adult CXRs (172 normal, 89 COVID-19 and 59 viral pneumonia) were included after applying predefined inclusion and exclusion criteria.

Inclusion criteria were patient age ≥ 18 years, availability of a frontal-view chest radiograph (posteroanterior or anteroposterior), and diagnostic image quality suitable for clinical interpretation. Exclusion criteria included pediatric patients, repeat or follow-up examinations from the same clinical episode, indeterminate diagnoses or mixed infections (e.g., bacterial co-infection), and images affected by severe motion artifacts or obscured lung fields.

Ground truth labels were established using a combination of laboratory results and clinical documentation. COVID-19 cases were defined by a positive reverse transcription polymerase chain reaction (RT-PCR) test for SARS-CoV-2 performed within 72 h of chest X-ray acquisition. Viral pneumonia cases were confirmed by virologic testing when available or in its absence by multidisciplinary clinical consensus based on presenting symptoms, laboratory markers, and radiological findings. COVID-19 ground truth was RT-PCR-confirmed, while viral pneumonia was based on virologic testing when available or clinical consensus, which may introduce label noise and potential circularity and could partly explain reduced viral pneumonia performance externally. Future work will prioritize larger lab-confirmed viral pneumonia cohorts and sensitivity analyses restricted to virologically confirmed cases. Normal cases were defined by a negative radiology report at the time of imaging and the absence of documented pulmonary pathology during a minimum follow-up period of seven days. All institutional images were independently reviewed by two board-certified chest radiologists. Discrepancies were resolved by consensus. Inter-rater reliability was quantified using Cohen’s kappa coefficient, demonstrating high agreement (κ = 0.89), consistent with recommendations for reliable ground-truth annotation in medical imaging studies [24]. This retrospective study was approved by the institutional ethics committee, and informed consent was waived due to anonymization of data.

### 2.3. Image Preprocessing and Data Augmentation

All chest X-ray images from both public and institutional datasets underwent standardized preprocessing to ensure consistency across sources and reduce the influence of non-clinical image characteristics. This approach was motivated by prior studies demonstrating that models trained on heterogeneous CXR datasets are susceptible to shortcut learning and confounding [22,23,24,25,26].

Images were resized to 224 × 224 pixels and pixel intensities were rescaled to the [0, 1] range. Each image was then normalized to zero mean and unit variance prior to network input. Resolution harmonization and contrast normalization were applied uniformly to mitigate scanner- and site-specific artifacts. To improve generalization and reduce overfitting, data augmentation was applied during training only. Augmentation strategies included random rotations (±15°), horizontal flipping, and random brightness adjustments (±10%). No augmentation was applied to validation or test datasets. Restricting augmentation to the training pipeline was intended to improve generalization without introducing distributional shifts into validation or external test evaluation.

### 2.4. Model Architecture

The classification model, termed AI-CXR.NET, was implemented using a convolutional neural network (CNN) with a ResNet-50 backbone pretrained on ImageNet [11]. ResNet-50 was selected due to its demonstrated performance and stability in medical imaging tasks compared with earlier CNN architectures such as VGG [10] and Inception [12]. In this study, we intentionally selected ResNet-50 as a widely validated and stable baseline backbone, pretrained on ImageNet, to allow a controlled evaluation of the study’s primary objectives: bias mitigation (harmonized preprocessing prior to dataset merging), patient-level cross-validation to reduce leakage, calibration assessment, and independent institutional external testing. Accordingly, this work was not designed as an architecture benchmarking study.

**Figure 1:**
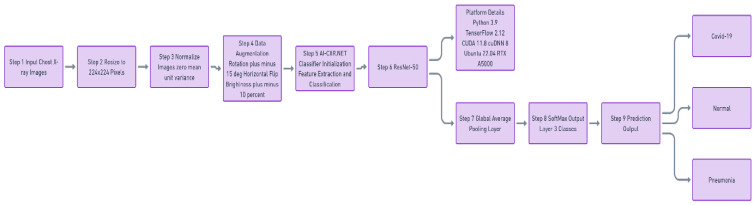
Workflow of the proposed AI-CXR.NET framework for chest X-Ray classification.

The network architecture consisted of the ResNet-50 feature extractor followed by a global average pooling layer and a fully connected SoftMax output layer producing class probabilities for normal, viral pneumonia, and COVID-19. The overall model workflow is illustrated in Figure 1.

### 2.5. Model Training and Optimization

Model training was performed using the Adam optimizer with an initial learning rate of 1 × 10^−4^, batch size of 32, and L2 regularization (weight decay) of 1 × 10^−5^. Training was conducted for a maximum of 25 epochs. Early stopping was implemented with a patience of three epochs based on validation loss to prevent overfitting. Model weights corresponding to the lowest validation loss were retained for subsequent evaluation, in line with established deep learning training practices [24]. All experiments were conducted using Python 3.9 and TensorFlow 2.12 on a workstation running Ubuntu 22.04 and equipped with an NVIDIA RTX A5000 GPU. CUDA 11.8 and cuDNN 8.x were used for GPU acceleration.

### 2.6. Cross-Validation Strategy and Reproducibility

To assess robustness and reduce sampling bias, stratified five-fold cross-validation was performed at the patient level within the public dataset. Each fold preserved the overall class distribution within ±1%, ensuring balanced representation of all diagnostic categories and minimizing the risk of data leakage. This approach follows established recommendations for preventing inflated performance estimates in medical imaging AI studies [24]. The distribution of patients across the five cross-validation folds is provided in Supplementary Materials (Table S1). In each cross-validation iteration, approximately 80% of the data was used for training and 20% for validation. All randomization processes—including fold assignment, batch ordering, data augmentation, and weight initialization—were conducted using a fixed random seed (42) to ensure full experimental reproducibility.

### 2.7. Performance Metrics and Statistical Analysis

Model performance was evaluated at the patient level using standard classification metrics, including accuracy, precision, recall (sensitivity), specificity, and F1-score (F1 = 2 × (Precision × Recall)/(Precision + Recall). Macro-averaged metrics were computed as the unweighted mean of class-wise values, while weighted metrics were calculated as the mean of class-wise values weighted by class support, consistent with methodological guidance for multi-class classification [24]. Ninety-five percent confidence intervals (95% CIs) were estimated using 1000 bootstrap replicates to assess metric stability. Confusion matrices were generated to summarize classification outcomes including true positives (TPs), true negatives (TNs), false positives (FPs), and false negatives (FNs).

Calibration of predicted probabilities was assessed using class-wise calibration curves (10 probability bins) and Brier scores (Supplementary Material S1). Brier scores were computed class-wise in a one-vs-rest (one-vs-all) fashion using the standard binary definition $\frac{1}{N}\sum_{i=1}^{N}(p_{i}-y_{i}{)}^{2}$, where $p_{i}$ is the predicted probability for the target class and $y_{i}\in (0,1)$ indicates class membership.

To explore potential clinical utility, decision curve analysis (DCA) was performed by comparing model-guided decision strategies with default “treat all” and “treat none” strategies across a range of threshold probabilities (0–1), as originally described by Vickers and Elkin [34] and recommended for evaluating clinical prediction models [27]. As part of future evaluation, we will report stratified performance by available acquisition metadata and perform stress tests designed to reduce or remove common shortcut cues (e.g., borders/markers and non-lung regions).

We conducted a precision–recall (PR) analysis on the independent institutional test dataset to assess class-wise performance under imbalanced conditions. The precision–recall curve, summarized using the average precision (AP) metric, was used to characterize the model’s ability to maintain precision across varying levels of recall. Precision–recall curves were computed in a one-vs-rest manner for each class, and AP was calculated as the area under each class-wise precision–recall curve on the independent institutional test dataset. Because AP can be sensitive to cohort size and class prevalence, we interpret very high AP values in conjunction with confusion matrices, confidence intervals, and an external dataset. Statistical analyses and visualizations were performed using SPSS (version 26.0) and Python-based scientific libraries.

For visualization of predicted probability patterns, per-image class probability outputs were retained for the validation and independent institutional test datasets. For each diagnostic category, the predicted probability corresponding to the ground-truth class was extracted on a per-image basis and summarized using box plots to illustrate differences in model confidence across datasets. This analysis was descriptive in nature and was not used for inferential or clinical interpretation.

## 3. Results

### 3.1. Model Training Dynamics and Cross-Validation Stability

**Figure 2:**
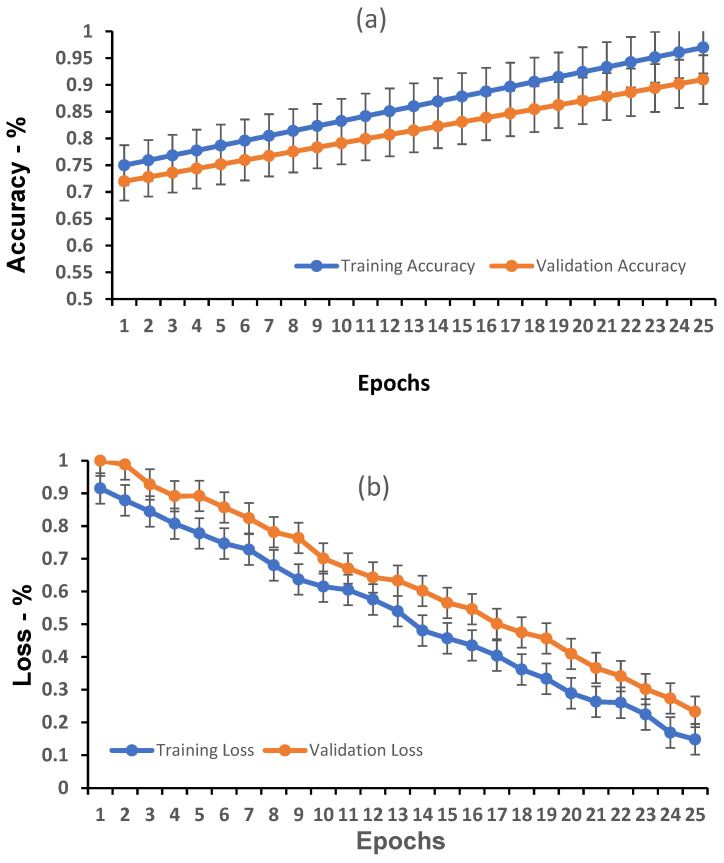
Model learning performance: (**a**) training and validation accuracy, (**b**) training and validation loss.

Learning curves for training and validation accuracy and loss are shown in Figure 2a,b. Training accuracy increased to approximately 97% at convergence, while validation accuracy reached approximately 91%**.** Correspondingly, training and validation losses decreased to approximately 0.15 and 0.25, respectively, indicating stable optimization without evidence of overfitting. The performance gap between the training and validation curves remained consistently small, suggesting effective learning without signs of overfitting.

### 3.2. Validation Dataset Classification

The confusion matrix for the training datasets is provided in the Supplementary Materials (Table S2) for completeness and is not discussed in the main text. The confusion matrix for the validation dataset is presented in Table 1, which illustrates the distribution of true positives, true negatives, false positives, and false negatives across all three diagnostic classes. Misclassifications were infrequent and occurred most commonly between viral pneumonia and COVID-19 classes.

On the validation dataset (n = 847), the model achieved an overall accuracy of 96.8% (95% CI: 95.4–97.8). Class-wise performance metrics, including precision, recall (sensitivity), specificity, and F1-score, are summarized in Table 2. Macro-averaged and weighted metrics exceeded 96% across all categories, demonstrating balanced performance despite class imbalance.

### 3.3. Predictive Values and Probability Distributions (Validation Dataset)

**Figure 3:**
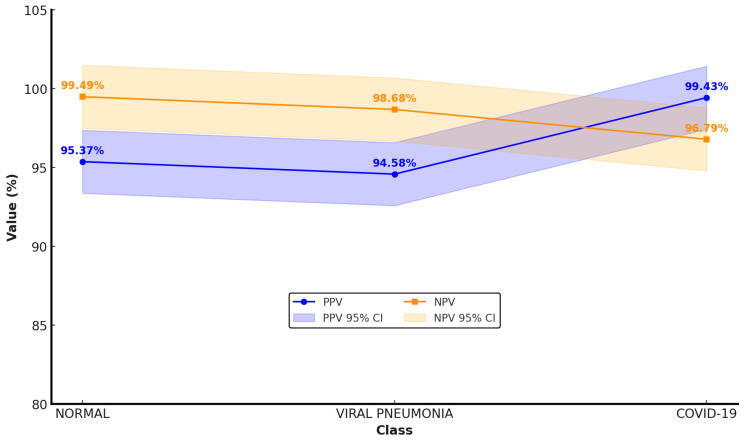
PPV and NPV with 95% confidence intervals (validation dataset).

Positive predictive values (PPVs) and negative predictive values (NPVs) were calculated for each diagnostic category on the validation dataset. High PPV and NPV values were observed across all classes, with NPVs exceeding 96% for normal and COVID-19 categories. Class-specific PPV and NPV estimates, along with 95% confidence intervals, are illustrated in Figure 3.

### 3.4. Calibration and Reliability Assessment (Validation Dataset)

**Figure 4:**
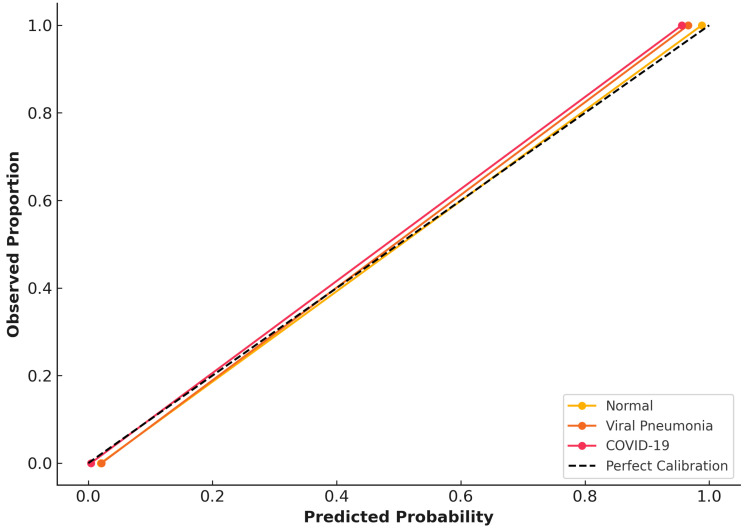
Class-wise calibration curves and probabilistic reliability on the validation dataset.

Class-wise calibration curves are shown in Figure 4, with predicted probabilities closely aligned with the ideal diagonal, reflecting well-aligned probabilistic estimates within this dataset. Brier scores were low across all diagnostic categories (normal: 0.0004, pneumonia: 0.0007, and COVID-19: 0.0008), reflecting well-aligned probabilistic estimates within this dataset. No systematic over- or under-confidence was observed within the validation cohort. However, we emphasize that calibration can degrade under domain shift, and we therefore interpret calibration results separately for the public validation and independent institutional datasets.

### 3.5. Decision Curve Analysis (Validation Dataset)

**Figure 5:**
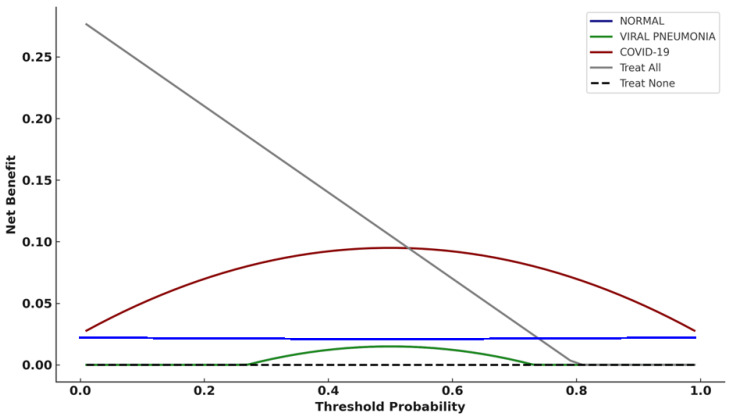
Decision curve analysis (DCA) comparing model predictions against default strategies on the validation dataset.

Decision curve analysis (Figure 5) indicated that, under hypothetical threshold assumptions, the COVID-19 class demonstrated exploratory net benefit patterns relative to default “treat all” and “treat none” strategies across a range of threshold probabilities. Viral pneumonia showed limited net benefit over narrower threshold intervals, while the normal class demonstrated minimal net benefit.

### 3.6. Performance on the Independent Institutional Test Dataset

The model was evaluated on the independent institutional test dataset from Adan Hospital (n = 320). Overall classification accuracy was 93.7% (95% CI: 90.54–95.92), representing a modest reduction relative to validation performance. This reduction is consistent with expected domain shift when models trained on public datasets are applied to real-world institutional data. The confusion matrix for the independent institutional test dataset is presented in Table 3.

Class-wise and aggregated performance metrics for the independent institutional test dataset are summarized in Table 4. Macro-averaged performance metrics exceeded 91% across all categories, indicating sustained multi-class performance despite reduced sample size and class imbalance. Misclassifications were most frequently observed between viral pneumonia and COVID-19 cases.

### 3.7. Predictive Values and Calibration (Institutional Dataset)

On the institutional dataset, PPV and NPV values remained high across all diagnostic classes. COVID-19 demonstrated an NPV exceeding 97%, supporting reliable exclusion of disease in negative cases. Class-specific PPV and NPV estimates are reported in Table 5. PPV and NPV values reflect dataset-specific prevalence and may vary under different clinical prevalence conditions.

**Figure 6:**
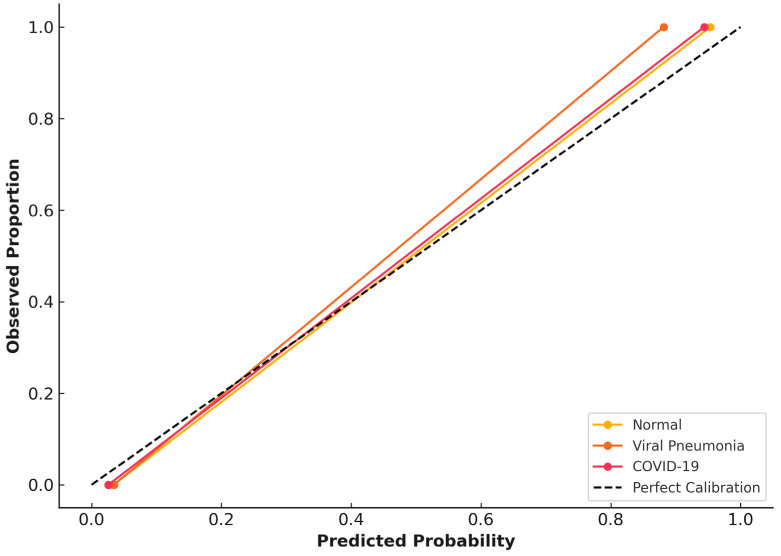
Class-wise calibration curves for the CNN model on the independent institutional test dataset.

Calibration curves for the institutional dataset are presented in Figure 6. Compared with validation data, calibration performance showed modest degradation, with slightly increased Brier scores (normal: 0.0017, viral pneumonia: 0.0035, and COVID-19: 0.0021) consistent with domain shift and reduced cohort size. Nevertheless, predicted probabilities remained well aligned with observed outcomes, with no evidence of systematic miscalibration.

### 3.8. Decision Curve Analysis (Institutional Dataset)

**Figure 7:**
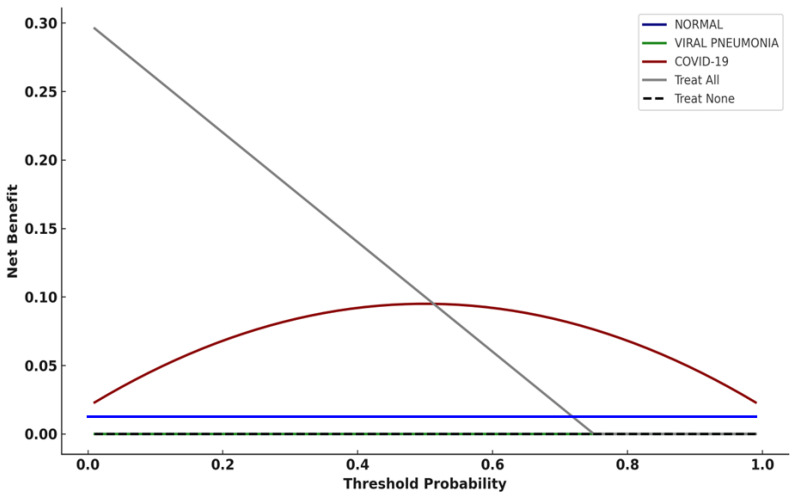
Decision curve analysis (DCA) for the CNN model applied to the independent institutional test dataset.

On the institutional dataset, similar exploratory net benefit patterns were observed for the COVID-19 class across moderate threshold probabilities, viral pneumonia and normal classes demonstrated limited or negligible net benefit without implying clinical effectiveness (Figure 7).

### 3.9. Prediction Distribution and Confidence Analysis

**Figure 8:**
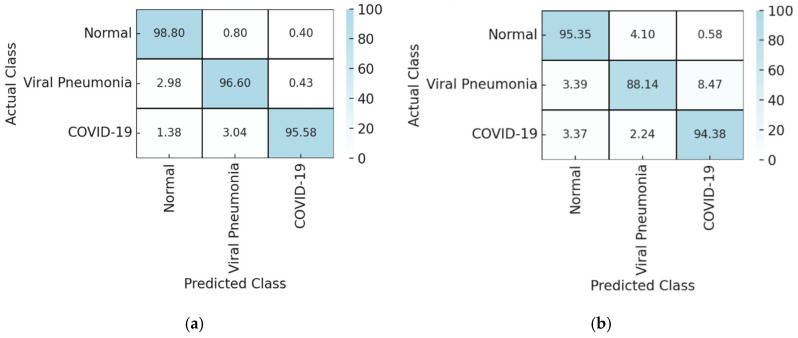
Class-wise prediction distribution heatmaps for: (**a**) validation and (**b**) the independent institutional test dataset.

Class-wise prediction distribution heatmaps across validation, and institutional test datasets are shown in Figure 8. These visualizations demonstrated high correct classification rates across datasets, with increased confusion between viral pneumonia and COVID-19 in the institutional cohort. The corresponding heatmap for the training dataset is provided in the Supplementary Materials (Figure S1).

**Figure 9:**
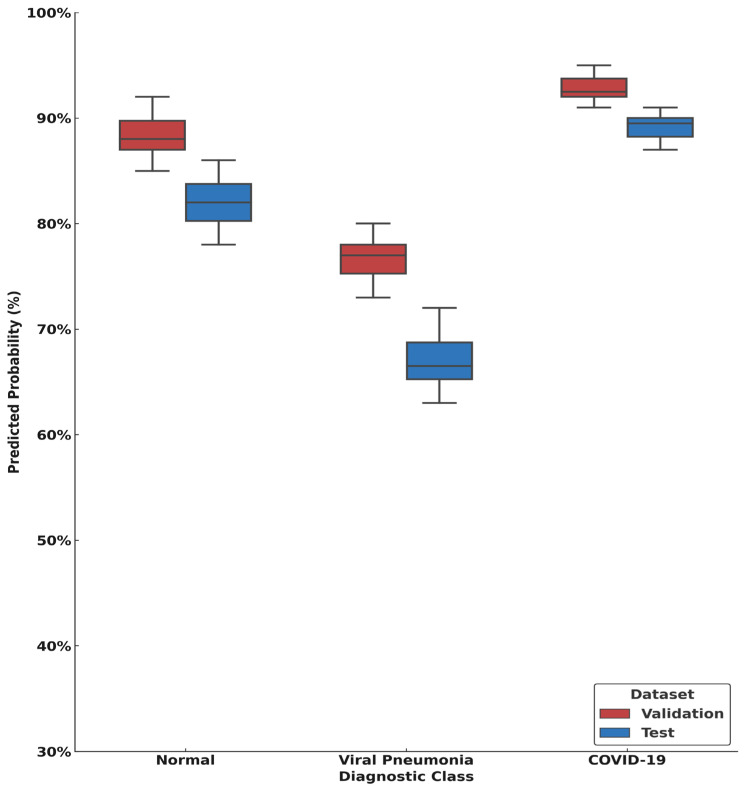
Box plots summarizing predicted class probability patterns for the validation dataset and the independent institutional test dataset across the three diagnostic categories.

Figure 9 presents box plots summarizing predicted class probability patterns for the validation and independent institutional test datasets. COVID-19 predictions exhibited comparatively narrower interquartile ranges across both datasets, whereas the normal and viral pneumonia classes showed broader interquartile ranges in the institutional cohort.

### 3.10. Precision–Recall Analysis (Institutional Dataset)

**Figure 10:**
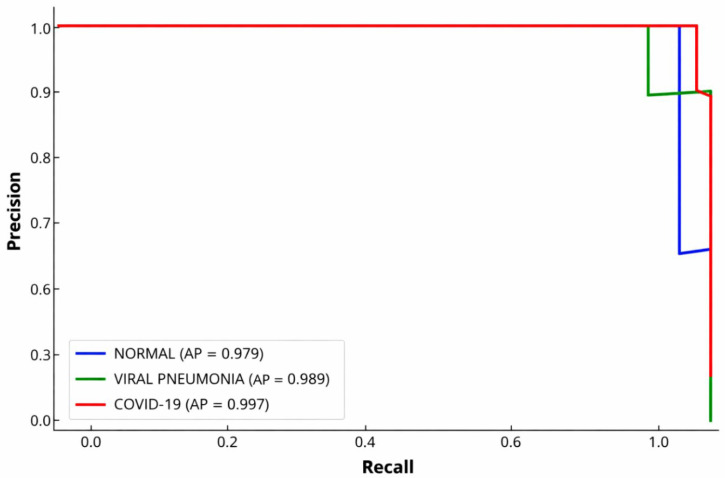
Precision–recall curves for normal, viral pneumonia, and COVID-19 classes on the independent institutional test dataset.

Figure 10 shows the precision–recall (PR) curves for the independent institutional test dataset. Average precision (AP) values were estimated for each class, and 95% confidence intervals were obtained using 1000 bootstrap resamples of the test dataset.

The COVID-19 class achieved the highest average precision (AP = 0.997, 95% CI: 0.992–0.999), followed by viral pneumonia (AP = 0.989, 95% CI: 0.980–0.995), and normal (AP = 0.979, 95% CI: 0.968–0.988). Across all classes, precision remained high over a broad range of recall values, with class-specific differences observed at higher recall levels.

## 4. Discussion

In this study, a convolutional neural network (CNN) was assessed for its effectiveness in classifying chest X-ray images into the following three distinct categories: normal, COVID-19, and non-COVID viral pneumonia. The evaluation encompassed the following three primary dimensions: the model’s ability to generalize across multiple datasets, the accuracy of its probability estimates (calibration), and its potential application in supporting clinical workflows. Notably, the model is not intended to serve as a standalone diagnostic tool.

Since radiographic patterns in different viral pneumonias often look similar, chest X-rays alone typically cannot reliably determine which virus is responsible. Therefore, any apparent differences between COVID-19 and other viral pneumonias on imaging may be influenced as much by how patient groups are selected and uncertainties in diagnostic standards as by actual disease-specific features [20,21]. For this reason, model class probabilities and thresholds should be viewed strictly as tools to support workflow decisions—not as definitive diagnoses. They may help prioritize reviews by radiologists, trigger confirmatory tests (such as repeat RT-PCR), guide isolation measures, or prompt further clinical evaluation according to local procedures, but they should not be the sole factor in making clinical or operational choices. Previous influential studies have highlighted that high AI performance for COVID-19 imaging sometimes results from unrelated clues and “shortcut learning”—like portable markers or location-specific artifacts—which can lead to overly optimistic results and poor generalization whenever there is dataset bias, noisy labels, or data leakage [25,26].

Robust discrimination metrics in non-specific imaging can be inflated by confounders like site, device, workflow, or patient case-mix instead of true disease features. Our use of patient-level partitioning, harmonized preprocessing, duplicate filtering, and independent institutional validation reduces—but does not eliminate—residual confounding. Thus, results serve as triage decision-support rather than conclusive evidence for etiologic discrimination. Large public datasets support model development but combining heterogeneous COVID-19 CXR sources risks misleadingly high results due to confounding, even with standardized preprocessing [22,25]. We interpreted performance on public validation cohorts cautiously and emphasized independent institutional testing.

On the public validation dataset (n = 847), the model demonstrated high overall accuracy and balanced class-wise performance, with narrow confidence intervals across evaluation metrics. When applied to an independent institutional dataset from Adan Hospital, performance declined modestly, reflecting real-world variability in imaging acquisition and case mix. External performance should be interpreted as initial single-center validation under domain shift, as transportability may vary with case-mix, acquisition practice (portable vs. fixed; AP vs. PA), device/vendor differences, and temporal drift in COVID-era labels. Prospective multicenter (including across-country) validation with stratified reporting is required. The largest external performance drop occurred in viral pneumonia, likely due to COVID–pneumonia overlap compounded by domain shift and greater label uncertainty relative to RT-PCR-confirmed COVID-19 (Table 4, Figure 8). High recall is crucial for infectious disease screening to avoid missing cases and limit transmission, while high precision and negative predictive value help use resources effectively and make safe rule-out decisions. Models trained on public datasets often underperform with real clinical data due to domain differences, diverse patients, and varied imaging methods, as found in prior studies [24,35]. Yet, consistent results across training, validation, and test groups—without major errors—demonstrate these models capture relevant clinical insights despite less certainty in practice.

Precision–recall (PR) analysis (Figure 10) revealed that when classes were imbalanced, COVID-19 had the highest average precision, followed by viral pneumonia and normal. This indicates better precision–recall for COVID-19 predictions in the independent dataset. Lower precision for other classes suggests more overlap among non-COVID pulmonary findings, demonstrating the need for evaluation metrics beyond accuracy in multi-class imaging.

This study found that negative predictive values (NPVs) were consistently high, especially for normal and COVID-19 cases in both validation and independent datasets. In radiology and emergency settings, high NPVs are crucial for safely excluding disease during triage, as false negatives can delay treatment and increase transmission risk (Table 5). Previous AI studies reported lower NPVs, with Wehbe et al. finding rates around 76–78% and Cohen et al. under 80% [35,36]. The higher NPVs in this study—especially for normal cases—suggest the models may effectively support screening and prioritization by quickly ruling out low-risk examinations, reducing workload and improving turnaround. Large-scale AI evaluations for normal CXRs reported similar benefits [19].

Calibration analysis showed strong alignment between predicted probabilities and actual outcomes on the validation set (Figure 4), with low Brier scores supporting reliability. Although calibration was slightly reduced on an external dataset (Figure 6), this aligns with expected effects from domain differences and sample size, highlighting the importance of local calibration before deployment. The model did not display consistent over- or under-confidence, indicating its probability estimates remain trustworthy in practice.

On both validation and institutional datasets, the COVID-19 class demonstrated positive net benefit over “treat all” and “treat none” strategies across moderate threshold ranges (Figure 5 and Figure 7). In this study, DCA does not define a single operational threshold; rather, it illustrates how in this study decision curve analysis (DCA) was used as an exploratory assessment based on hypothetical threshold assumptions; also, it illustrates how net benefit could vary under hypothetical thresholds if model outputs were used for triage-oriented actions such as prioritizing radiologist review, triggering repeat confirmatory testing (e.g., RT-PCR) or isolation precautions according to local protocols, or escalating evaluation in clinically appropriate cases. Threshold selection and downstream actions are site-specific and depend on prevalence, capacity constraints, and the relative harms of false-positive versus false-negative triage decisions; therefore, prospective workflow studies are required before clinical implementation.

In contrast, the viral pneumonia and normal classes demonstrated limited or negligible net benefit. This pattern reflects the limited clinical value of intervention decisions based solely on CXR-based etiologic predictions and highlights the importance of selecting appropriate clinical use cases. As emphasized in methodological guidance [27,34], DCA results should be interpreted in conjunction with clinical context, disease prevalence, and downstream consequences of decision thresholds.

Overall, AI-CXR.NET may provide supportive signals for triage-oriented workflows under appropriate clinical governance and continued human oversight. However, results should be interpreted cautiously in light of confounding risks, label uncertainty, and domain shift, and should be validated prospectively within clearly defined clinical pathways.

### Limitations and Future Directions

Several limitations warrant emphasis. First, the independent institutional test dataset was derived from a single center, which limits generalizability to hospitals with different case-mix, acquisition practices (portable vs. fixed; AP vs. PA), and workflows; where acquisition metadata permit, future work will report stratified performance (e.g., AP vs. PA; portable vs. fixed) to better characterize domain shift. Second, the viral pneumonia subgroup in the institutional cohort was small (n = 59), which increases uncertainty in class-specific estimates and likely contributed to the lower precision observed for viral pneumonia on external testing (85.6%) and greater confusion with COVID-19; thus, these findings should be interpreted as initial external validation under real-world domain shift, not evidence of multi-site transportability, and larger, balanced multicenter cohorts are needed. Third, although we applied multiple safeguards to reduce leakage and dataset bias in public-data training, residual confounding cannot be fully excluded without dedicated shortcut audits and sensitivity analyses [18,19,20,21,22]. While preprocessing, patient-level partitioning (where feasible), and independent external testing were used to mitigate shortcut learning, we did not perform targeted shortcut audits (e.g., marker/border or non-lung masking tests) or attribution-based visualizations (e.g., Grad-CAM) to directly assess reliance on non-pathological cues; these will be added in future work. Fourth, only ResNet-50 was evaluated; future work will benchmark newer CNN and Transformer backbones (e.g., DenseNet, EfficientNet-V2/ConvNeXt, and ViT) under identical preprocessing and using the same independent institutional test set for fair comparison. Fifth, etiologic separation between COVID-19 and other viral pneumonias on CXR is inherently constrained by overlapping radiographic patterns, reinforcing the need for prospective evaluation within clearly defined triage pathways. Finally, clinical utility has not yet been assessed in real workflows or reader studies; upcoming work will prioritize multicenter prospective evaluation (e.g., silent deployment) and multi-reader, multi-case studies to quantify effects on triage prioritization, safety (including false-negative rates), and efficiency.

## 5. Conclusions

This study developed and externally evaluated a ResNet-50-based deep learning model for classifying chest radiographs as normal, COVID-19, or non-COVID viral pneumonia, with emphasis on generalization, calibration, and triage-oriented decision support. The results indicate that a carefully developed CXR classifier can maintain useful performance when transferred from public datasets to an independent real-world hospital cohort, while producing probabilistic outputs that can be evaluated for reliability. Importantly, the contribution of this work is not a claim of etiologic diagnosis from radiography, but a more transparent and clinically cautious evaluation approach—combining bias-aware dataset handling, independent external validation, and calibration-focused reporting—to support workflow triage use cases. The observed performance decline on external data underscores that prospective multicenter validation across diverse sites and time periods is required before clinical adoption. Overall, these findings suggest potential utility for prioritization and rule-out support under appropriate clinical governance, but they do not establish clinical effectiveness; prospective studies linked to clearly defined downstream actions and safety endpoints remain essential before any routine clinical deployment.

## Figures and Tables

**Table 1:** Confusion matrix for the validation dataset (n = 847).

Actual\Predicted	Normal	Viral Pneumonia	COVID-19	Total
Normal	247	2	1	250
Viral pneumonia	7	227	1	235
COVID-19	5	11	346	362
Total	259	240	348	847

**Table 2:** Class-wise and aggregated classification metrics for the validation dataset.

Class	Precision %	Recall	Specificity	F1-Score %	Support
Normal	95.37 (92.1–97.3)	98.8 (96.5–99.6)	97.9 (96.5–98.8)	97.1	250
Viral pneumonia	94.6 (90.9–96.8)	96.6 (93.4–98.3)	97.88 (96.4–98.7)	95.6	235
COVID-19	99.43 (97.9–99.8)	95.58 (92.94–97.3)	99.59 (98.5–99.9)	97.5	362
Weighted average	96.9	96.8	98.6	96.8	
Macro average	96.4	96.9	98.5	96.7	

CI = confidence interval. Values in parentheses indicate 95% confidence intervals estimated using 1000 bootstrap replicates.

**Table 3:** Confusion matrix for the independent institutional test dataset (n = 320).

Actual\Predicted	Normal	Viral Pneumonia	COVID-19	Total
Normal	164	7	1	172
Viral pneumonia	2	52	5	59
COVID-19	3	2	84	89
Total	169	61	90	320

**Table 4:** Class-wise and aggregated classification metrics for the independent institutional test dataset.

Class	Precision %	Recall %	Specificity %	F1-Score %	Support
Normal	97.0 (93.3–98.7)	95.6 (91.1–97.6)	96.6 (92.3–98.6)	96.2	172
Viral pneumonia	85.6 (74.3–92.0)	88.1 (77.5–94.1)	96.6 (93.6–98.2)	86.7	59
COVID-19	93.3 (86.2–96.9)	94.4 (87.5–97.6)	97.4 (94.5–98.8)	93.8	89
Weighted average	93.8	93.7	96.8	93.8	
Macro average	91.9	92.6	96.8	92.2	

Values in parentheses indicate 95% confidence intervals estimated using 1000 bootstrap replicates.

**Table 5:** Predictive values for the independent institutional test dataset.

Class	PPV %	NPV %
Normal	97.0 (93.3–98.7)	94.7 (90.9–97.2)
Viral pneumonia	85.2 (74.3–92.0)	97.3 (94.6–98.9)
COVID-19	93.3 (86.2–96.9)	97.8 (95.1–99.1)

PPV: positive predictive value; NPV: negative predictive value. Values in parentheses indicate 95% confidence intervals estimated using 1000 bootstrap replicates.

## Data Availability

The data are not publicly available due to Ministry of Health (Kuwait) patient confidentiality regulations. Data supporting the findings of this study are available from the corresponding author upon reasonable request and subject to institutional approval.

## Supplementary Materials

The following supporting information can be downloaded at https://www.mdpi.com/article/10.3390/diagnostics16070995/s1.

## Abbreviations

CNNs	Convolutional Neural Networks
CXR	Chest X-rays
AI-CXR.NET	Artificial Intelligence Chest X-ray Network
NPVs	Negative Predictive Values
PPVs	Positive Predictive Values
AP	Average Precision
PR	Precision–Recall
DCA	Decision Curve Analysis
RSNA	Decision Curve Analysis
NIH	National Institute of Health
PHC	Primary Health Care

## References

[1] Shelke A., Gajbhiye M., Kshirsagar P. (2021). Chest X-ray classification using deep learning for automated COVID-19 screening. SN Comput. Sci..

[2] Al Nufaiei Z.F., Alshamrani K.M. (2025). Comparing Ultrasound, Chest X-Ray, and CT Scan for Pneumonia Detection. Med. Devices.

[3] Khan E., Rehman M.Z.U., Ahmed F., Alfouzan F.A., Alzahrani N.M., Ahmad J. (2022). Chest X-ray classification for the detection of COVID-19 using deep learning techniques. Sensors.

[4] Central Statistical Bureau (CSB) (2023). Demographic Indicators 2023.

[5] Esteva A., Kuprel B., Novoa R.A., Ko J., Swetter S.M., Blau H.M., Thrun S. (2017). Dermatologist-level classification of skin cancer with deep neural networks. Nature.

[6] Saxena A., Singh S.P. (2022). A deep learning approach for the detection of COVID-19 from chest X-ray images using convolutional neural networks. arXiv.

[7] Gouda W., Almurafeh M., Humayun M., Jhanjhi N.Z. (2022). Detection of COVID-19 based on chest X-rays using deep learning. Healthcare.

[8] Chowdhury M.E.H., Rahman T., Khandakar A., Mazhar R., Kadir M.A., Bin Mahbub Z., Islam K.R., Khan M.S., Iqbal A., Al Emadi N. (2020). Can AI help in screening viral and COVID-19 pneumonia?. IEEE Access.

[9] Rahman T., Khandakar A., Qiblawey Y., Tahir A., Kiranyaz S., Kashem S.B.A., Islam M.T., Al Maadeed S., Zughaier S.M., Khan M.S. (2021). Exploring the effect of image enhancement techniques on COVID-19 detection using chest X-ray images. Comput. Biol. Med..

[10] Simonyan K., Zisserman A. (2014). Very deep convolutional networks for large-scale image recognition. arXiv.

[11] He K., Zhang X., Ren S., Sun J. (2016). Deep residual learning for image recognition. Proceedings of the IEEE Conference on Computer Vision and Pattern Recognition (CVPR).

[12] Szegedy C., Liu W., Jia Y., Sermanet P., Reed S., Anguelov D., Erhan D., Vanhoucke V., Rabinovich A., Liu W. (2015). Going deeper with convolutions. Proceedings of the IEEE Conference on Computer Vision and Pattern Recognition (CVPR).

[13] Alom M.Z., Taha T.M., Yakopcic C., Westberg S., Sidike P., Nasrin M.S., Hasan M., Van Essen B.C., Awwal A.A.S., Asari V.K. (2019). A State-of-the-Art Survey on Deep Learning Theory and Architectures. Electronics.

[14] Karhan Z., Kal F.A. (2020). COVID-19 classification using deep learning in chest X-ray images. Proceedings of the Medical Technologies Congress (TIPTEKNO).

[15] Asif S., Wenhui Y., Hou J., Jinhai S. (2020). Classification of COVID-19 from chest X-ray images using deep convolutional neural networks. Proceedings of the IEEE 6th International Conference on Computer and Communications (ICCC).

[16] Pala M.A., Navdar M.B. (2025). SPX-GNN: An Explainable Graph Neural Network for Harnessing Long-Range Dependencies in Tuberculosis Classifications in Chest X-Ray Images. Diagnostics.

[17] Anderson P.G., Tarder-Stoll H., Alpaslan M., Keathley N., Levin D.L., Venkatesh S., Bartel E., Sicular S., Howell S., Lindsey R.V. (2024). Deep learning improves physician accuracy in the comprehensive detection of abnormalities on chest X-rays. Sci. Rep..

[18] Caliman Sturdza O.A. (2025). Deep Learning Network Selection and Optimized Information Fusion for Enhanced COVID-19 Detection: A Literature Review. Diagnostics.

[19] Kumar A., Patel P., Robert D., Kumar S., Khetani A., Reddy B., Srivastava A. (2024). Accuracy of an artificial intelligence-enabled diagnostic assistance device in recognizing normal chest radiographs: A service evaluation. BJR Open.

[20] Wang G., Liu X., Shen J. (2021). A deep-learning pipeline for the diagnosis and discrimination of viral, non-viral and COVID-19 pneumonia from chest X-ray images. Nat. Biomed. Eng..

[21] Wong H.Y.F., Lam H.Y.S., Fong A.H.T., Leung S.T., Chin T.W.-Y., Lo C.S.Y., Lui M.M.-S., Lee J.C.Y., Chiu K.W.-H., Chung T.W.-H. (2020). Frequency and distribution of chest radiographic findings in COVID-19-positive patients. Radiology.

[22] Oakden-Rayner L. (2020). Exploring large-scale public medical image datasets. Acad. Radiol..

[23] Maguolo G., Nanni L. (2021). A critical evaluation of methods for COVID-19 automatic detection from X-ray images. Inf. Fusion.

[24] Roberts M., Driggs D., Thorpe M., Gilbey J., Yeung M., Ursprung S., Aviles-Rivero A.I., Etmann C., McCague C., Beer L.L. (2021). Common pitfalls and recommendations for using machine learning to detect and prognosticate for COVID-19 using chest radiographs and CT scans. Nat. Mach. Intell..

[25] DeGrave A.J., Janizek J.D., Lee S.-I. (2021). AI for radiographic COVID-19 detection selects shortcuts over signal. Nat. Mach. Intell..

[26] Geirhos R., Jacobsen J.-H., Michaelis C., Zemel R., Brendel W., Bethge M., Wichmann F.A. (2020). Shortcut learning in deep neural networks. Nat. Mach. Intell..

[27] Collins G.S., Reitsma J.B., Altman D.G., Moons K.G.M. (2015). Transparent reporting of a multivariable prediction model for individual prognosis or diagnosis (TRIPOD): The TRIPOD Statement. Ann. Intern. Med..

[28] Collins G.S., Moons K.G.M., Dhiman P., Riley R.D., Beam A.L., Van Calster B., Ghassemi M., Liu X., Reitsma J.B., van Smeden M. (2024). TRIPOD+AI statement: Updated guidance for reporting clinical prediction models that use regression or machine learning methods. BMJ.

[29] Moons K.G., Damen J.A., Kaul T., Hooft L., Navarro C.A., Dhiman P., Beam A.L., Van Calster B., Celi L.A., Denaxas S. (2025). PROBAST+AI: An updated quality, risk of bias, and applicability assessment tool for prediction models using regression or artificial intelligence methods. BMJ.

[30] Rahman T., Khandakar A., Qiblawey Y., Tahir A., Kiranyaz S., Abul Kashem S.B., Islam M.T., Al Maadeed S., Zughaier S.M., Khan M.S. (2021). COVID-19 Radiography Database. https://www.kaggle.com/datasets/tawsifurrahman/covid19-radiography-database.

[31] Cohen J.P., Morrison P., Dao L., Roth K., Duong T., Ghassem M. (2020). COVID-19 image data collection: Prospective predictions are the future. arXiv.

[32] Wang X., Peng Y., Lu L., Lu Z., Bagheri M., Summers R.M. (2017). ChestX-ray8: Hospital-scale chest X-ray database and benchmarks on weakly supervised classification and localization of common thoracic diseases. Proceedings of the IEEE Conference on Computer Vision and Pattern Recognition (CVPR).

[33] Radiological Society of North America (RSNA) (2018). RSNA Pneumonia Detection Challenge. https://www.kaggle.com/competitions/rsna-pneumonia-detection-challenge.

[34] Vickers A.J., Elkin E.B. (2006). Decision curve analysis: A novel method for evaluating prediction models. Med. Decis. Mak..

[35] Murphy K., Smits H., Knoops A.J.G., Korst M.B.J.M., Samson T., Scholten E.T., Schalekamp S., Schaefer-Prokop C.M., Philipsen R.H.H.M., Meijers A. (2020). COVID-19 on Chest Radiographs: A Multireader Evaluation of an Artificial Intelligence System. Radiology.

[36] Cohen J.P., Dao L., Roth K., Morrison P., Bengio Y., Abbasi A.F., Shen B., Mahsa H.K., Ghassemi M., Li H. (2020). Predicting COVID-19 pneumonia severity on chest X-ray with deep learning. Cureus.

