# Supplementary material for: Externally Validated Deep Learning Analysis of Chest Radiographs for Differentiating COVID-19 and Viral Pneumonia
Source: Diagnostics (Basel). 2026 Mar 26;16(7):995. doi: 10.3390/diagnostics16070995 (PMC13072745; doi:10.3390/diagnostics16070995)

Supplement materials:

Table S1. Distribution of patients per diagnostic category (normal, viral pneumonia, and COVID-19) across the five stratified cross-validation folds used for model training and internal validation.

Fold	Normal (n)	Viral Pneumonia (n)	COVID-19 (n)	Total (n)	Normal %	Viral Pneumonia %	% COVID-19
Fold 1	500	377	579	1 456	34.3	25.9	39.8
Fold 2	501	376	579	1 456	34.4	25.8	39.8
Fold 3	499	378	578	1 455	34.3	26.0	39.7
Fold 4	500	377	579	1 456	34.3	25.9	39.8
Fold 5	500	375	578	1 453	34.4	25.8	39.8
Mean ± SD	500 ± 1	377 ± 1	579 ± 1	1 455 ± 1	34.34 ± 0.04	25.88 ± 0.08	39.78 ± 0.05

Values are reported as absolute counts. Class proportions were preserved within ±1% across folds.

Table S2. Confusion matrix for the training dataset (n = 7276)

Actual \ Predicted	Normal	Viral Pneumonia	COVID-19	Total
Normal	2470	20	10	2500
Viral pneumonia	36	1 775	72	1 883
COVID-19	37	73	2 783	2 893

Supplementary material S1. Brier score calibration

In this study, Brier scores were computed class-wise in a one-vs-rest fashion. For each class, we used the standard binary Brier score definition:

$$\text{Brier} = \frac{1}{N}\sum_{i=1}^{N}(p_i - y_i)^2,$$

where p_i is the model-predicted probability for that class and $y_i \in \{0,1\}$ indicates whether the instance belongs to that class (1) or not (0). We report these class-wise Brier scores alongside the calibration curves using probability binning (B =10 bins), to provide a scalar summary of probabilistic accuracy complementary to visual calibration assessment.

Figure S1. The corresponding heatmap for the training dataset.

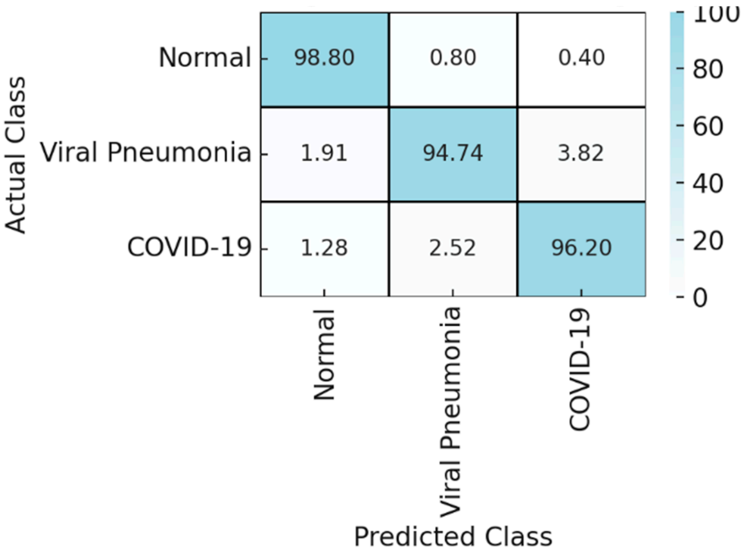

Supplement: Supplementary file 1 [file diagnostics-16-00995-s001.zip › diagnostics-4160995-supplementary.pdf]
